# Mouse sepsis models: don't forget ambient temperature!

**DOI:** 10.1186/s40635-022-00457-4

**Published:** 2022-07-01

**Authors:** Dario Lucas Helbing, Leonie Karoline Stabenow, Reinhard Bauer

**Affiliations:** Institute of Molecular Cell Biology, Jena University Hospital, Jena, Germany

The recently published contribution by Bauer et al. [1] in this journal deciphers the heuristic value of a biological definition of sepsis as a failing stress response. Rightly, the authors proposed to expand the concept of sepsis by incorporating infectious stress within the general organismic stress response to define sepsis as an illness state characterized by allostatic overload and failing adaptive responses along with biotic (pathogen) and abiotic environmental (e.g., ambient temperature, *T*_a_) stress factors.

We want to take the opportunity to ventilate a serious shortcoming of experimental sepsis research, which compromises its translational value: the systematic disregard of *T*_a_ on sepsis progression and outcome (Table 1). Textbook knowledge says that thermoregulation is a fundamental homeostatic function of all mammals. It includes afferent thermal sensing, central regulation, and an efferent response with the consequence of tightly controlled body temperature within a narrow species-specific range [2]. Variations of core body temperature (*T*_c_) outside this range trigger autonomic thermoregulatory responses, mainly via a gradually increased sympathetic activity [3]. Clinical data clearly indicate that spontaneous *T*_c_ lowering (hypothermia indicating energy exhaustion) is directly correlated with poor outcome of sepsis [4–6]. Hence, in clinical settings, recommendations clearly define optimal ambient temperature ranges for appropriate care of septic patients to prevent cold stress. Therefore, T_a_ is controlled within a narrow range of thermoneutral temperatures at which energy expenditure to maintain body temperature is lowest to save metabolic demands and prevent additional cold stress and its negative consequences on critically ill patients [7].

**Table 1:** Ambient temperatures in experimental sepsis research with mouse models (2019–2022)

Temperature ranges	20–26°C	29–32°C	> 32 °C	Not specified	Not verified
	SHTLM	TNZM	OHTR		
*Sepsis induced by:*					
Bacterial infection	108	1	0	314	7
Viral infection	1	0	0	20	
Fungal infection	2	0	0	17	
Parasitic infection	12	0	0	31	
(Severe systemic inflammation)	120	2	0	235	14

Results of a literature research derived from a PubMed search with restriction to time of publication (2019–2022) but not language using EndNote X9 and keywords: sepsis and mouse and in vivo. (*SHTLM* standard housing temperatures for laboratory mice, *TNZM* thermoneutral zone for mice, *OHTR* overheating temperature range.)

Surprisingly, this fundamental prerequisite to warrant best possible care for patients is widely unregarded in experimental sepsis research. Mice exhibit a rather unfavorable surface area to body mass ratio as well as an unfavorable whole body thermal conductance (> one order of magnitude difference between mouse and human): therefore, already under healthy and thermoneutral conditions (similar for mouse and human at ~ 30 °C), mice have to compensate it by an enhanced basal metabolic rate.

Incredibly, almost all sepsis experiments with mice are done at “room temperature”! However, these standard housing temperatures for laboratory mice, e.g., *T*_a_ of 20 °C and 24 °C [8–10] induce chronic cold stress for mice. Healthy mice are capable of controlling such a challenge and maintain their core temperature through an appropriate increase of their metabolism. Indeed, energy demand is increased by about 50% at *T*_a_ of 22 °C compared with thermoneutral conditions [11]. However, sick mice are compromised by severe infection or systemic inflammation and additional cold stress markedly altered their response to sepsis by recruiting different defense mechanisms. Recently, two seminal publications appeared on experimental sepsis research and mild cold stress. Ganeshan et al. [12] exemplified that activation of immunity (by LPS and bacterial sepsis) causes an energetic trade-off with homeothermy (the stable maintenance of core temperature), resulting in hypometabolism and hypothermia. Among other measures, the primary outcome parameter was survival. Sepsis caused by induced bacteremia led to an increased mortality rate in mice kept at *T*_a_ = 30 °C (~ 40%) compared with mice kept at *T*_a_ = 22 °C (~ 5%). A similar result in terms of mortality rate was reported in response to LPS at the dosages used there (LPS 1–1.5 mg/kg): in mice kept at *T*_a_ = 30 °C (~ 75%) compared with mice kept at *T*_a_ = 22 °C (~ 50%). In contrast, Carpenter et al. [13] reported that the survival rate of male C57BL/6 mice housed at 30 °C (78%) after abdominal sepsis was significantly increased compared with mice housed at 22 °C (40%). This is in line with findings after LPS administration [14]. These findings highlight the pronounced impact of housing temperature on established sepsis models and the importance of reporting housing temperature.

All in all, these studies substantiate the importance of tightly controlling *T*_a_ to prevent significant bias in results from preclinical animal research on infection and inflammation. Hence, a decisive difference is whether cold-adapted (stressed) mice or mice housed under thermoneutral (unstressed) conditions were characterized to be “well-designated mouse models”, particularly when translational implications are addressed. Accounting for *T*_a_ will likely improve the predictive power and value of preclinical sepsis research and may aid in overcoming the “replication crisis” [15].

We strongly recommend considering thermoneutral conditions as “standard housing conditions for mice in translational approaches” to improve animal modeling in sepsis [16].
